# Refugees’ caring and commoning practices against marginalisation under COVID‐19 in Greece

**DOI:** 10.1111/1745-5871.12522

**Published:** 2021-11-22

**Authors:** Charalampos Tsavdaroglou, Maria Kaika

**Affiliations:** ^1^ Department of Human Geography, Planning and International Development (GPIO), AISSR University of Amsterdam Amsterdam The Netherlands

**Keywords:** campisation, caringscapes, commoning, COVID‐19, Greece, refugees

## Abstract

This article documents and juxtaposes two side effects of the COVID‐19 pandemic on refugee health, housing, and living conditions in Greece. First is the intensification of state‐led practices of what is increasingly known as “campisation,” hyper‐isolation, and ultimately the stigmatisation of refugee populations. Second is the intensification of refugee‐led “commoning” practices of self‐ and community care and the creation of “caringscapes” inside and outside the camps, which has produced new sociospatial connections that have challenged isolation. Documenting these interrelated processes side by side, we draw attention to two important insights. First is that the proliferation of caringscapes acts as an important, but ultimately insufficient, antidote against increased exclusion marginalisation and stigmatisation of refugees. Second is that new ethics and new forms of collective care that have emerged alongside repeated mantras about individual responsibility and social distancing can become levers to imagine a less individualistic, less divisive, and less isolated world.

## Graphical abstract

This article documents and juxtaposes two “side effects” of the COVID‐19 pandemic on refugee health, housing, and living conditions in Greece. First is the intensification of state‐led practices of campisation, hyper‐isolation, and ultimately stigmatisation of refugee populations. Second is the intensification of refugee‐led commoning practices of self‐ and community care and the creation of “caringscapes” inside and outside the camps that produced new sociospatial connections that challenged isolation.

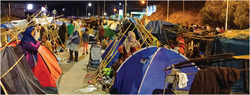

**Key insights**

In this article, we bring to the fore two parallel processes of the COVID‐19 pandemic on refugee health, housing, and living conditions in Greece. On one hand, we critically reflect on the state policies of campisation and stigmatisation of refugee populations. On the other hand, we explore caring and commoning practices enacted by refugees. Contextualizing these two conflicting and interrelated processes, we introduce a novel concept, *refugees' caringscapes*, which act against state policies of fencing and marginalisation.

## 1 INTRODUCTION

Today we announce 27 new cases of the coronavirus in our country and 23 additional cases in the Refugee Accommodation Structure in Ritsona.

(press release, Greek National Public Health Organization, 2020, 2 April)

The excerpt above comes from one of the official public updates issued daily by the Greek government in the early months of the COVID‐19 pandemic. The text shows how discrimination between citizens and refugees/asylum seekers was intensified and became more strongly institutionalised during the pandemic. Like many other countries, the Greek state issued numerous strict policies of severe movement restriction, prompting citizens to “stay at home” and practice social/physical distancing to stay safe. These policies attracted both praise and criticism and have become the focus of scholarly attention (COVID‐19_Regional_Labour, 2020; Leontidou, 2020). However, little research has thus far been published on the impacts that these strict measures and lockdowns have had on the institutional and public treatment of refugees and asylum seekers and on how they have affected their health, housing, and living conditions.

In this article, we show how the hyper‐isolation of refugees and asylum seekers inside camps during the pandemic in Greece was seen as a means to keep “safe” members of society outside the camps that led to increased health risks for refugee and asylum seeking populations. The campaign to “stay home [in order to] stay safe” [*Μένουμε Ασφαλείς ‐Μένουμε σπίτι*] that the Greek government released in March 2020 was completely nonsensical when applied to overcrowded state‐run refugee camps or overcrowded private refugee housing in city centres. We argue that this campaign and the pandemic exposed the stark inadequacy of refugee housing provision inside and outside camps and exacerbated institutional and individual practices and discourses of marginalisation and stigmatisation.

We also document commoning practices of self‐ and community caring developed and performed by refugees and asylum seekers during the pandemic in response to their increased institutional marginalisation and as a way to manage the increased health risks that the pandemic posed. Activist and NGO‐led practices of care for and solidarity with refugees emerged during the pandemic across Europe and have received academic attention (Chatzidakis et al., 2020; Milan, 2020; Springer, 2020). Yet, there is little documentation about or analysis of the refugees’ own self‐caring and self‐organised commoning practices in response to inadequate state support for them during the pandemic.

By documenting side by side these two interrelated processes, we show how the ethics and forms of commoning and collective care that emerged in refugee communities provide an antidote to marginalisation and to mantras about individual responsibility and social/physical distancing that dominated public discourse during the pandemic. These commoning practices of self‐ and community care were by no means sufficient to fence off the pandemic or to significantly raise living standards among refugees in camps. But they could potentially act as levers to ignite an international dialogue by which to imagine a less individualistic, less divisive, and less isolated world.

The article draws upon material from ethnographic fieldwork conducted at 10 state‐run refugee camps in Athens, Thessaloniki, and Lesvos between March and September 2020. We conducted 30 semi‐structured interviews with refugees who were residents of camps at the time. All refugees who participated in the research are adults and their names in quotes below have been altered in order to protect their identities.

## 2 STAYING AT HOME: A DANGEROUS ACT INSIDE AND OUTSIDE CAMPS

Greece is at the epicentre of the European refugee crisis and one of the main migratory routes into Europe. During the last five years, more than one million newcomers from the Middle East and Africa have entered and crossed the country to reach central and north European countries (UNHCR, 2020). After the 2016 common statement between the European Union (EU) and Turkey (European Council, 2016), the so‐called “Balkan route” closed for refugees and asylum seekers, and thousands found themselves “trapped” in Greece. Today, it is estimated that more than 115,000 refugees and asylum seekers live in Greece (UNHCR, 2020). The vast majority are settled in overcrowded state‐run camps located far from city centres, often on lands designated officially as industrial zones and therefore inappropriate for housing.

Many humanitarian organisations and scholars have been critical of the poor living conditions evident in the camps (Greek Council for Refugees, 2019; International Rescue Committee, 2016; Tsavdaroglou et al., 2019; Vradis et al., 2019). They have argued that camps do not fully abide by UN Habitat (2009) or EU (ECRE, 2007) principles for security of tenure, availability of services, materials, facilities and infrastructure, habitability, and accessibility. However, the pandemic brought a new dimension to the problems related to inadequate refugee housing; namely, the overcrowded living conditions in state‐run camps meant that the health of those in camp populations could not be adequately protected from infections and outbreaks. As reported by 24 humanitarian organisations:

Conditions [in the camps] cannot be characterized as suitable for dignified humane living. Extremely limited access to running water, toilets, and showers, as well as hours‐long lines for food distribution and insufficient medical … personnel, make it impossible to abide by the guidelines for protection from the coronavirus. (Action Aid Hellas et al., 2020)

The International Federation of Red Cross (IFRC), International Organization for Migration (IOM), United Nations High Commissioner for Refugees (UNHCR), and World Health Organization (WHO) also drew attention to the living and health conditions of refugees, and in a joint statement asked governments to take into account that:

people … living in camps … [are] faced with specific challenges … that must be taken into consideration when planning for … response operations for the COVID‐19 outbreak. They are frequently neglected, stigmatized, and may face difficulties in accessing health services that are otherwise available to the general population. (IFRC et al., 2020, p. 2)

The first COVID‐19 lockdown in Greece started on 23 March and ended on 4 May 2020, with relatively few cases and deaths. Elementary and secondary schools reopened on 1 June, and the country fully opened its borders to tourists on 15 June 2020. However, the government kept in place the quarantine and restriction measures in state‐run refugee camps until 31 August 2020. That means that people in refugee camps were forced to observe quarantine and lockdown measures for an additional four months compared with Greek citizens and an additional two and a half months compared with tourists visiting Greece.

In the words of a teacher in the refugee state‐run camp of Koutsohero in central Greece:

This decision is not justified on the basis of public health … on the contrary, it increases [the refugees’] vulnerability to the pandemic as it traps them in unacceptable living conditions … This discriminatory attitude of the Greek state also causes the exclusion of refugee children from education. Schools have reopened [in Greece], but refugee children are not allowed to return to them. [Participant, Kosmopoulou, 2020]

Several humanitarian organisations have drawn attention both to the intensification of the isolation of refugees in camps during the pandemic and to the alarming deterioration of living conditions that came with it. The Hebrew Immigrant Aid Society (HIAS, 2020a, p. 1) highlighted that according to “the lack of adherence to … COVID‐19 guidelines [in camps] … it is anticipated that a single COVID‐19 case could lead to hundreds … of otherwise preventable deaths.” Médecins Sans Frontières (2020a) pleaded with the Greek government to evacuate the refugee camps during the pandemic, arguing that:

COVID‐19 should not be used as a tool to detain … refugees. We continue to call for the evacuation of people… from the [camps] to safe accommodation [since camps] … become even more perilous pits of violence, sickness, and misery when people are unable to move due to arbitrary restrictions.

It should be noted here that the measures of isolation in state‐run refugee camps also included cutting individual financial assistance provided by the state to prevent refugees from visiting areas outside the camps. As the lawyers of HIAS (2020b) have observed, refugees were often fined with the 150 euros fee for “unnecessary travel during a lockdown,” even when they visited city centres in search of legal assistance.

All evidence supports the view that hyper‐isolation and fencing of refugees in state‐run camps was strengthened during the COVID‐19 pandemic. This condition is close to what Kreichauf (2018, p. 14) terms campisation, the development of territorial zones “for the purpose of separating the ‘own’ and the ‘(ethnic) stranger;’ citizens and non‐citizens; [and for creating] exceptional places to house this particular group and not citizens.” The sociospatial practices involved in campisation normalise conditions of exception and exclusion, since camps “are legally under the jurisdiction of the host society but also exempted from it” (Turner, 2016, p. 141). Considering what Wacquant (2007, p. 67) calls regimes of marginality, we argue that during the pandemic Greece’s state‐run refugee camps constituted a paradigmatic expression of policies of marginalisation and stigmatisation.

But refugees in state‐run camps were not the only people discriminated against and strongly affected by state policies during the pandemic. Those who had been granted asylum status and had been living in state‐paid housing in cities with monthly income support were also affected by changes to policies during the period between March and August 2020. Specifically, the Greek government announced in May 2020 that around 10,000 people granted asylum status and living in state‐paid housing in cities would be evicted and their cash assistance cards would be blocked with effect from 1 June (Médecins Sans Frontières, 2020b). This action went against an international protocol that underlines that “protection against forced evictions is a key element of the right to adequate housing” (UN Habitat (2009, p. 4). It brought many people under immediate and serious threat of homelessness at a time when “staying at home” was the key policy for “staying safe.” The policy also increased the stigmatisation of refugees as high‐risk COVID‐19 carriers. Dozens of testimonies in the media reported such stigmatisation on public transport and in public spaces. One of the few attempts by Ministry of Migration and Asylum to rehouse vulnerable refugees (mostly women and children) from island state‐run camps to a hotel in Arnissa Pellas in North Greece was met with violent opposition by a few locals who set the hotel on fire, arguing that “the coronavirus is just a diversion tactic; they lock us inside our houses in order to transfer illegal immigrants to our cities” (Alterthess, 2020). This serious incident is characteristic of how the pandemic intensified underlying and multi‐layered dimensions of sociospatial stigmatisation, marginalisation, and campisation of refugees.

## 3 SELF‐ AND COMMUNITY CARING VERSUS INDIVIDUAL RESPONSIBILITY

Faced with increased isolation and health risk in camps, many refugees organised protests to improve living conditions in state‐run camps at the beginning of the pandemic. Many fled the camps illegally. But many who stayed trapped inside the camps during the lockdown initiated a range of novel practices of self‐ and community care.

Many scholars have emphasised the point that the camp is not “a static place” (Kreichauf, 2018, p. 4; see also Martin, 2015; Ramadan, 2009; Sanyal, 2014); rather, it is a political space, comprising multiple interrelations, conflicts, and complexities. Our research confirms this conjuncture. During the pandemic, we witnessed the birth of several humanitarian organisations and services, and of self‐organised “coronavirus awareness” and self‐caring groups and practices inside camps, all of it in the context of diminishing state support. For example, the “White Helmets” group formed in the Moria refugee camp on Lesvos Island. The group took its name from the “White Helmets” group in Syria, whose members had helped people trapped in bombed‐out buildings, and saw itself as “an independent group of Syrian and Arab refugees … created … during the Corona Crisis to help all refugees in Moria camp” (White Helmets, 2020). During the lockdown, it organised cleaning and sanitation shifts as well as teaching activities in the camp.

Another group called the “Moria Corona Awareness Team” (Moria Corona Awareness Team, 2020) was also created in the camp by “teachers, pharmacists, and other professionals of many nationalities” to provide various kinds of help (Human Rights Watch, 2020). As a member of the team explained, “all our members are from inside the camp … our responsibility is to inform people about coronavirus; we should take care of ourselves” (Human Rights Watch, 2020).

The groups also set up a face mask factory, in which over 50 refugees from Syria, Afghanistan, Eritrea, and Congo who used to work as tailors in their home countries made and distributed inside the camp 300 face masks, free of charge. As Mohammad from Afghanistan explained to us, “the conditions were out of control so we knew we needed to do something ourselves” (1 August 2020). “It is remarkable,” Fatima from Syria then told us, “that refugees care for themselves without almost any hygiene provisions by state authorities” (1 August 2020). It is important to note that the production of face masks by refugees also takes place in other camps on the Greek islands and around mainland Greece. These practices demonstrate that non‐hierarchical and self‐organised practices of care, mutual aid, and sharing are possible even under desperate conditions of existence.

In addition, a refugee community centre called Café Patogh (Café Catch Up), which has operated in the heart of Athens since 2019, remained open during the 2020 pandemic, distributing daily meals wrapped in individual plastic bags. Named hope bags, they were offered to refugees who were homeless during that period. People from the community centre also organised meals on wheels, a food distribution system for homeless people in different areas of Athens. In the words of one group member, “Our house is a community which stands in solidarity with humans in need—particularly the homeless and the refugees. During the COVID‐19 lockdown we self‐organised meals for the most vulnerable people of the city” (5 August 2020).

In parallel with these direct and positive initiatives of self‐organisation and care, there have been several protest actions, particularly in state‐run camps across the country. These events enabled participants to object to the refugees’ hyper‐isolation and restricted movements and to the announced evictions. Many demonstrations were followed by violent riot police operations in camps on Chios and Samos islands and in Ritsona camp in Athens. Refugees also occupied the Vagiohori camp in North Greece for five days in protest against the isolation and the threat of homelessness. In Kranidi in Peloponnese in South Greece, refugees protested media stigmatisation, broke the police cordon of isolation, and exited the camp. Refugees in deportation centres in Athens and Paranesti in North Greece organised hunger strikes against overcrowded conditions.

**FIGURE 1:**
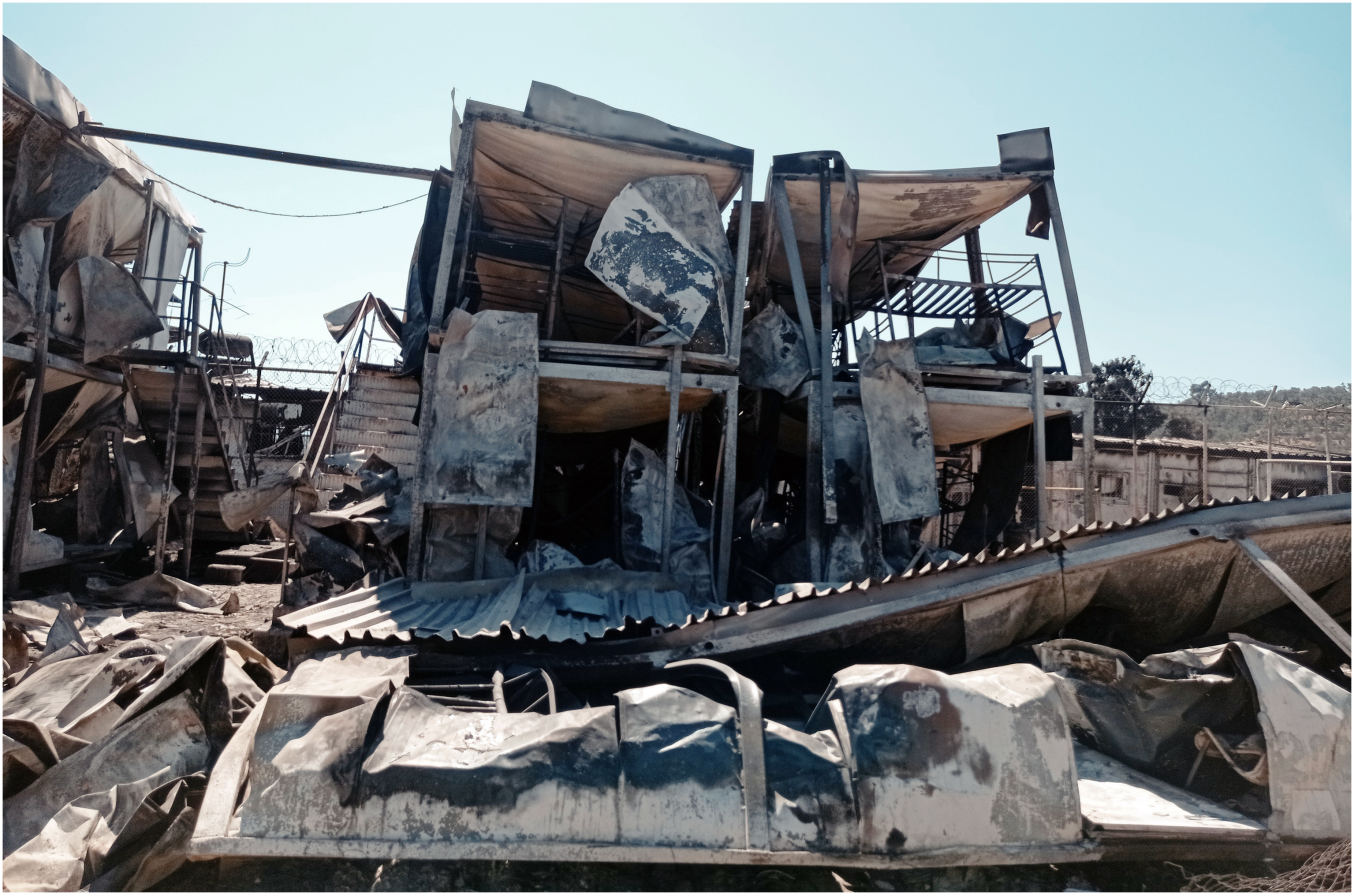
The burned Moria refugee camp in Lesvos Island. Source: the authors

Then the Moria refugee camp on Lesvos Island was burnt down by refugees protesting confinement measures (Figure 1). Moria had been purpose‐built in 2013 to accommodate around 3,000 people, but by the September of 2020 it held around 20,000 people. Although restrictive measures and the lockdown against COVID‐19 had been lifted for all Greek citizens on 4 May 2020 and Lesvos opened for tourists on 1 July, the Moria camp remained in lockdown throughout the summer. On 8 September, amidst continuing mobility restrictions and strict quarantine measures in the camp, refugees set the camp on fire and escaped to nearby areas.

**FIGURE 2:**
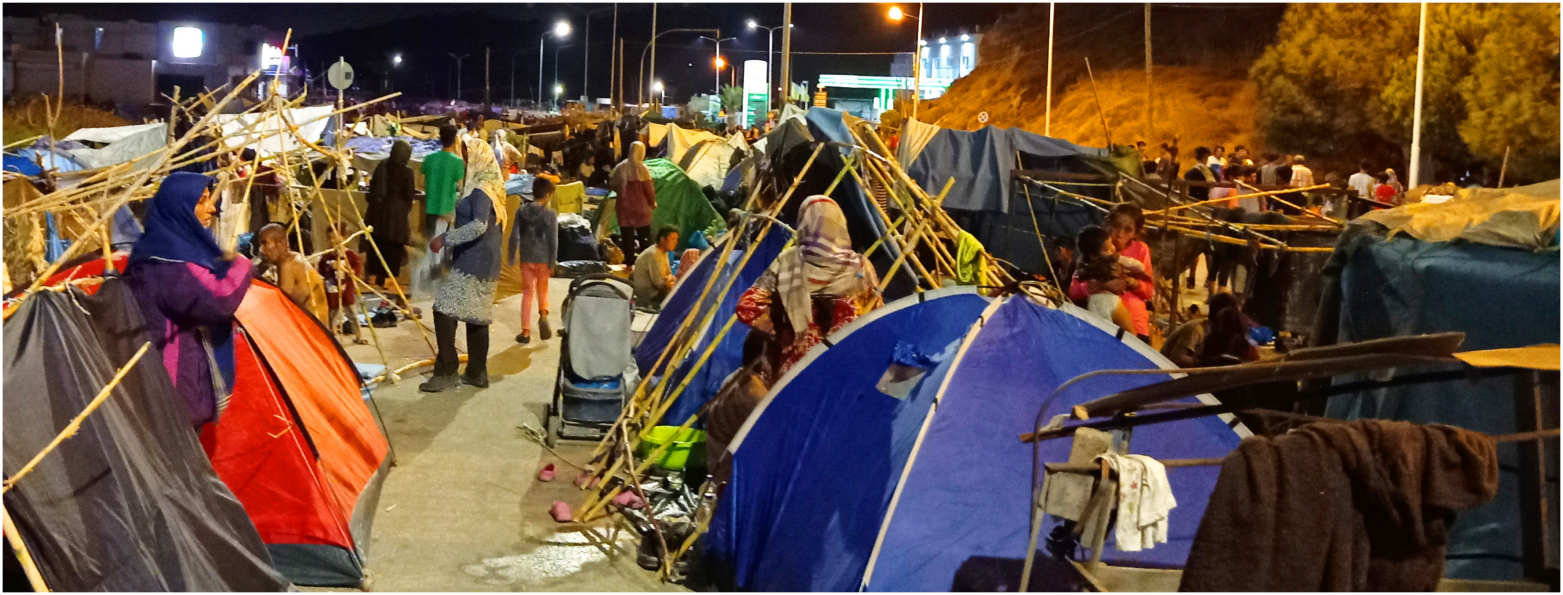
Refugees’ makeshift settlement on the road to Mytilene. Source: the authors

During the two weeks following the fire, thousands of refugees created makeshift settlements on the road that connects Moria to Mytilene, the Island’s capital city (Figure 2). Karima from Syria stressed that “all together, Syrians, Afghans, and Africans took care of each other, irrespective of country of origin or religion … we wanted to escape from the hell of Moria camp and we did it all together” (12 September 2020). Riots and protests followed the camp’s fire and after 15 days the police violently enclosed the refugees in a new campsite with inadequate health and housing provision.

## 4 CARINGSCAPES AND COMMONING IMAGINARIES

In recent years, scholars in geography, anthropology, and urban studies have focused on care and caring practices (see, for example, Green et al., 2014). Feminist geographers have suggested care ethics as “not so much an activity [but also] as a way of relating to others” (McEwan & Goodman, 2010, p. 103; see also Held, 2006; Staeheli & Brown, 2003). Chatzidakis et al. (2020, n.p.) have stressed the sociopolitical aspect of care, suggesting that it refers to “our ability to provide the political, social, material, and emotional conditions that allow for the vast majority of people and living creatures on this planet to thrive—along with the planet itself.” Valenzuela’s (1999, p. 109) treatment of care as “deliberately bringing issues of race, difference, and power into central focus” is a useful approach to the focus of this article, namely, the study of refugees’ caring practices during the COVID‐19 pandemic.

Many newly emerging practices of mutual care adhere to the principle of the commons and commoning (Tsavdaroglou & Kaika, 2021; Williams, 2018) because they are non‐hierarchical and based on self‐governance. Many scholars have emphasised how such open commoning practices differ significantly from state‐led or private‐led practices of care that often involve physical and social enclosures (Dellenbaugh et al., 2015; Jeffrey et al., 2012; Tsavdaroglou, 2018). That argument is particularly important for our study on refugees’ self‐organised activities in response to state policies of campisation in Greece. Important also for our inquiry is an analysis by De Angelis (2003, p. 1) of the commons as “necessarily created and sustained by ‘communities’ … by social networks of mutual aid, solidarity, and practices of human exchange.”

Calling these social practices “commoning” is a neologism introduced by Linebaugh (2008, p. 279) to stress the point that “the commons is an activity and, if anything, it expresses relationships in society [and thus] … it might be better to keep the word as a verb, an activity, rather than as a noun.” An increasing number of scholars have been engaging with the idea that commoning is interconnected with practices of care or have proposed that care is a key element of social relations of commoning (Moore, 2018; Tsavdaroglou et al., 2019). As Parris and Williams (2019, p. 534) have noted, “for commons to be sustained and maintained, practices of care are crucial to their continued existence.” Likewise, Gibson‐Graham et al. (2013, p. 147) have argued that there is an urgent need for people to become commoners, in order to shape “the ways in which we are accessing, using, benefiting from, caring for, and taking responsibility for commons.”

Primary and secondary forms of evidence analysed in this article support arguments advanced by Trimikliniotis et al. (2015) and Papadopoulos and Tsianos (2013, p. 179), who contend that commoning is clear in instances of “shared knowledge, affective cooperation, mutual support and care between migrants when they are on the road or when they arrive somewhere.” In the context of the current pandemic, Stavrides (2020, n.p.) also prompts us to think if “life is both the source and the scope of the common in the prospect of an emancipated society.” On such grounds, we argue that bringing into dialogue debates on commoning and those on care can illuminate refugees’ experiments with commoning activities to create caring communities as practices that respond to discriminatory state enclosures and policies of quarantine and campisation during the COVID‐19 pandemic. In this respect, and as argued by Gibson‐Graham et al. (2013, p. 138), “the practice of commoning is motivated by an ethic of care.” Refugees’ activities discussed in sections above demonstrate a unique ethos of caring central to solidarity commoning practices (Tsavdaroglou et al., 2019). Thus, we also argue that one key characteristic of such self‐caring practices, which is also parallel to commoning, is that they produce novel spaces and social networks that are akin to caringscapes. The notion of caringscapes was proposed to express the informal interdependencies across different dimensions of the human life course (McKie et al., 2002). We suggest that refugees’ self‐caring practices highlight the potentiality of an *emergent commoning caringscape* beyond and against the tendencies of the state and the market to cut off any expression of self‐organised relation and practice. Refugees’ self‐caring collective practices amidst the pandemic and against institutional campisation show that commoning across borders of race, religion, and ethnicity is possible.

## 5 CONCLUSION

Historically, pandemics have forced humans to break with the past and imagine their world anew. This one is no different. It is a portal, a gateway between one world and the next. (Roy, 2020)

Hyper‐isolation in Greek state‐run refugee camps during the COVID‐19 pandemic intensified physical and social borders for refugees and increased their stigmatisation and marginalisation. However, the commoning practices of self‐ and community care that refugees enacted during the same period signalled an opening and a possibility to make a “chink in the wall” (Papadopoulos & Tsianos, 2013, p. 179) and destabilise existing barriers.

Refugees are not the only people who intensified practices of care during the pandemic. The Care Collective (2020, n.p.), an initiative that emerged during the pandemic, has proposed a care manifesto underpinned by the concept of caring communities, and has noted that “if the pandemic has taught us anything so far, it is that we are in urgent need of a politics that puts care front and centre of life.” Springer (2020, p. 112) has also recently documented the proliferation of caring collectives under the pandemic, emphasising the importance of the caring geographies of mutual aid linked to “the failings of both capitalism and the state.”

In response, we highlight three key points that also signal the possibility for opening new imaginaries of commoning and practices of care against the enclosures and individualisation that the pandemic brought.

First, although the pandemic became an excuse to normalise and intensify refugees’ sociospatial distancing and exceptional state of marginality and isolation (campisation by any other term), at the same time the pandemic ignited unexpected and ad hoc practices of solidarity and mutual care and aid that challenged those newly created or intensified sociospatial boundaries. Second, although mundane, the everyday practices of self‐ and community care that refugees established during the pandemic give reason to reignite a discussion on the commons. Even though the emergent commoning and caring collectives of refugees did not abolish camps as such, they nevertheless produced new spaces and new sociospatial relations inside the camps that changed their functions dramatically; they created new caringscapes, spaces of interrelations, connectivity, tenderness and, in general, spaces that have offered the possibility for being‐in‐common even within states of marginality and isolation. Third, the commoning and caring practices that refugees enacted during the pandemic went against the rhetoric and practice of individual responsibility that became the main official policy guideline against the pandemic in most countries. In the midst of repeated mantras about responsibility and social/physical distancing, new forms of collective commoning and acts of care emerged among the most vulnerable population groups.

The ethos of collective caring that emerged out of these processes, the ways in which they generated new commoning practices, and the ways in which they broke existing national and religious divides among refugees, all confirm that “life itself is an intricate and beautifully complex web of mutual aid relations” (Springer, 2020, p. 113). These practices can be a lever to imagine a less divisive, less isolated, and bordered world.

## CONFLICT OF INTEREST

The authors declared no potential conflicts of interest with respect to the research, authorship, and/or publication of this article.

## FUNDING INFORMATION

The authors disclosed receipt of the following financial support for the research, authorship, and/or publication of this article: This scientific publication is an outcome of the RE‐HOUSING project, which has received funding from the European Union’s Horizon 2020 research and innovation programme under the Marie‐Skłodowska Curie (grant agreement no. 795992).

## ETHICS STATEMENT

Hereby, I, Charalampos Tsavdaroglou, consciously assure that for the manuscript *Refugees’ caring and commoning practices against marginalisation under COVID‐19 in Greece*, the following are fulfilled:

1. This material is the authors’ own original work, which has not been previously published elsewhere.
2. The paper is not currently being considered for publication elsewhere.
3. The paper reflects the authors’ own research and analysis in a truthful and complete manner.
4. The paper properly credits the meaningful contributions of co‐authors.
5. The results are appropriately placed in the context of prior and existing research.
6. All sources used are properly disclosed. Literal copying of text is indicated as such by using quotation marks and giving proper reference.
7. All authors have been personally and actively involved in substantial work leading to the paper, and will take public responsibility for its content.

## Data Availability

No datasets were generated or analysed during the current study.
